# The oral KIF11 inhibitor 4SC‐205 exhibits antitumor activity and potentiates standard and targeted therapies in primary and metastatic neuroblastoma models

**DOI:** 10.1002/ctm2.533

**Published:** 2021-10-14

**Authors:** Marc Masanas, Nuria Masiá, Leticia Suárez‐Cabrera, Mireia Olivan, Aroa Soriano, Blanca Majem, Laura Devis‐Jauregui, Rebeca Burgos‐Panadero, Carlos Jiménez, Pau Rodriguez‐Sodupe, Ariadna Boloix, Ignasi Toledano, Gabriela Guillén, Alexandra Navarro, David Llobet‐Navas, Alberto Villanueva, Josep Sánchez de Toledo, Josep Roma, Rosa Noguera, Lucas Moreno, Rolf Krauss, Soledad Gallego, Anna Santamaria, Miguel F. Segura

**Affiliations:** ^1^ Group of Translational Research in Child and Adolescent Cancer Vall d'Hebron Research Institute (VHIR) ‐ Universitat Autònoma de Barcelona (UAB) Barcelona Spain; ^2^ Cell Cycle and Cancer Laboratory Biomedical Research Group in Urology Vall d'Hebron Research Institute (VHIR) ‐ Universitat Autònoma de Barcelona (UAB) Barcelona Spain; ^3^ Translational Oncology Laboratory Anatomy Unit Department of Pathology and Experimental Therapy School of Medicine Universitat de Barcelona (UB) L'Hospitalet de Llobregat Spain; ^4^ Molecular Mechanisms and Experimental Therapy in Oncology‐Oncobell Program Bellvitge Biomedical Research Institute (IDIBELL) L'Hospitalet de Llobregat Spain; ^5^ Group of Translational Research in Pediatric Solid Tumors Department of Pathology Medical School University of Valencia‐INCLIVA Biomedical Health Research Institute Valencia Spain; ^6^ Low Prevalence Tumors. Centro de Investigación Biomédica en Red de Cáncer (CIBERONC) Instituto de Salud Carlos III Madrid Spain; ^7^ Quantitative Genomic Medicine Laboratory qGenomics Barcelona Spain; ^8^ Department of Surgery Universitat Autònoma de Barcelona (UAB) Barcelona Spain; ^9^ Department of Pathology Vall d'Hebron University Hospital Universitat Autònoma de Barcelona Barcelona Spain; ^10^ Group of Chemoresistance and Predictive Factors Subprogram Against Cancer Therapeutic Resistance (ProCURE) ICO Oncobell Program IDIBELL L'Hospitalet del Llobregat Barcelona Spain; ^11^ Xenopat S.L., Business Bioincubator Bellvitge Health Science Campus Barcelona Spain; ^12^ Catalan Institute of Oncology (ICO) Barcelona Spain; ^13^ Pediatric Oncology and Hematology Department Hospital Universitari Vall d'Hebron ‐ Universitat Autònoma de Barcelona (UAB) Barcelona Spain; ^14^ 4SC Martinsried Germany

Dear Editor,

Neuroblastoma remains incurable for most patients with high‐risk disease.^1^ Perturbation of transcription factors (MYCN and PHOX2B), kinases (ALK, MEK), and cell cycle regulators (CDK4/6, CHECK1), among other factors, make neuroblastoma cells highly proliferative, which is associated with poor patient outcomes.^2^, ^3^ To circumvent the limitations of the classical microtubule poisons such as vinca alcaloyds used in the treatment of neuroblastoma,^1^ we sought to explore alternative mitotic regulators as new therapeutic targets for high‐risk neuroblastoma patients. One of these mitotic spindle‐specific proteins is kinesin family member 11 (KIF11), also known as kinesin spindle protein, kinesin‐5, or Eg5, which is essential for bipolar spindle formation and mitotic progression in human cells.^4^

Transcriptomic analyses showed that the expression of multiple kinesins, including *KIF11* was higher in the high‐risk neuroblastoma compared with low‐ and intermediate‐risk groups (Figures 1A and S1; Table S1). Overall survival was significantly poorer in patients with high *KIF11* expression (Figure 1B–D; Table S2). *KIF11* high expression was identified as an independent prognostic factor of survival, together with risk assessment (HR = 3.051; Table S3) and found to be higher in patients with amplification of *MYCN*, 1p36 loss, or 17q23 gain (Figure 1E). At the protein level, KIF11 expression was detected in the cytoplasm of neuroblastic cells (Figure 1F) and showed higher expression compared to low‐ or intermediate‐risk neuroblastoma samples (*p* < 0.05) and in tumors with segmental chromosome alterations such as 1p36, 11q deletion, and gain of 17q23 (Table S4). Kaplan–Meier analysis confirmed that high KIF11 protein expression was associated with shorter event‐free and overall survival (Figures 1G and H). While there is a positive correlation between *KIF11* and *MYCN* mRNA expression levels, MYCN is neither sufficient, nor necessary for KIF11 expression (Figure S2).

**FIGURE 1:**
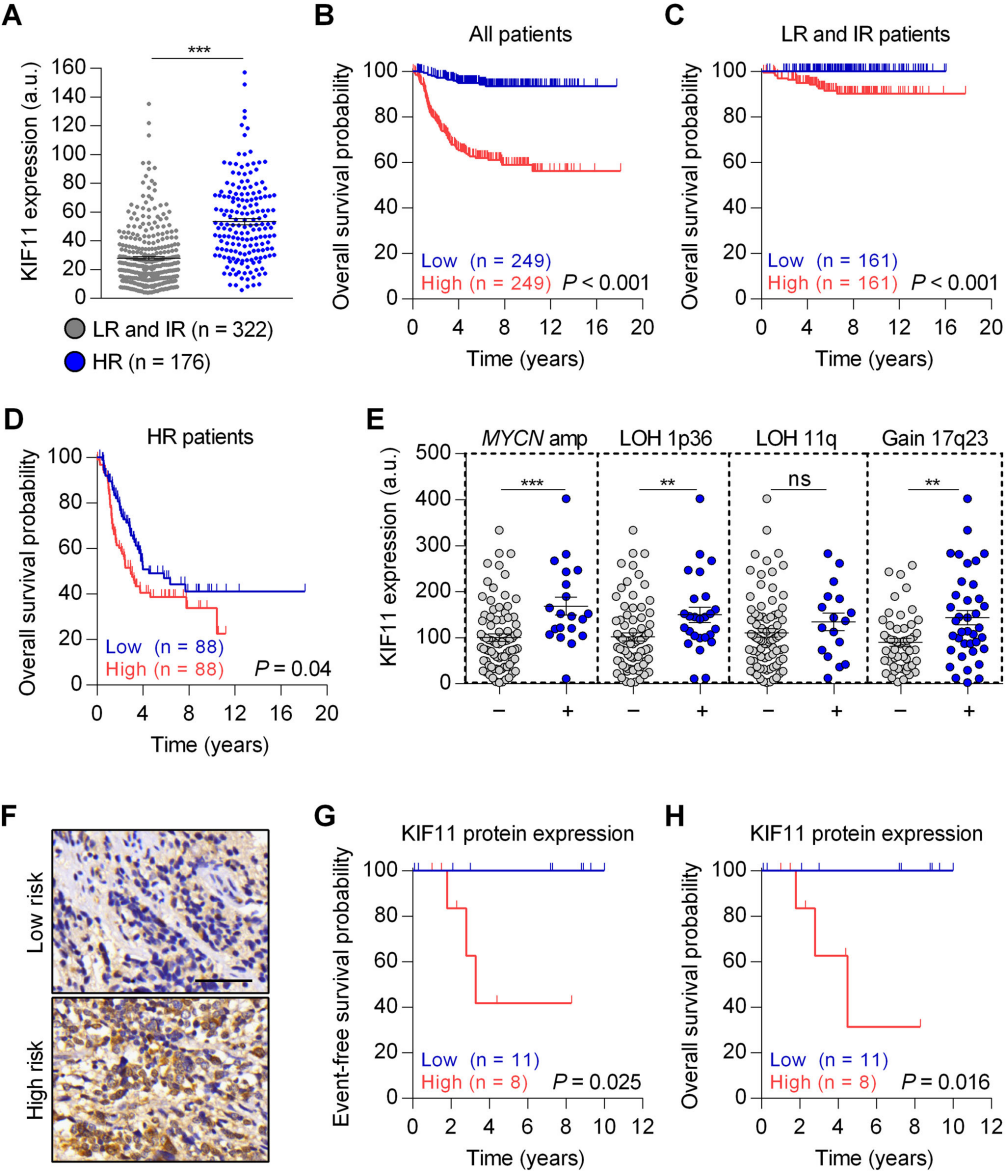
KIF11 expression is an independent prognostic factor of survival in neuroblastoma. (A) *KIF11* mRNA expression levels comparing low‐/intermediate‐ with high‐risk neuroblastoma tumors (GSE62564, *n* = 498). (B‐D) Kaplan–Meier overall survival curve in a cohort of 498 patients based on *KIF11* mRNA expression (B) or stratified in low‐ and intermediate‐risk (C) or high‐risk (D) neuroblastoma subcohorts. (E) *KIF11* mRNA expression in neuroblastoma patients with different genomic alterations (GSE3960, *n* = 101). (F) Representative images of KIF11 immunohistochemistry in low‐and high‐risk neuroblastoma tissues. Scale bar indicates 50 μm. (G and H) Kaplan–Meier curves of event‐free survival (G) and overall survival (H) based on KIF11 protein expression

According to functional genomics, neuroblastoma cells seem to be one of the cell types that are more dependent on the expression of KIF11 for survival being particularly sensitive to its pharmacological inhibition.^5^ Concurring with these observations, the silencing of KIF11 caused a reduction in cell viability (Figure S3A‐C) and a 3–4 fold reduction in the growth of established neuroblastoma subcutaneous xenografts (Figure 2A–C and S3D‐R). KIF11 inhibitors have moved forward toward phase 1 and 2 clinical trials in adult tumors,^6^, ^7^ with very limited development for childhood cancer. Herein, we provide a complete preclinical characterization of the potent and highly selective KIF11 inhibitor, 4SC‐205 (Figure 2D), the first oral KIF11 inhibitor that has been evaluated in phase I clinical trials in adult patients (NCT01065025). Compared to other KIF11 inhibitors, 4SC‐205 can be administrated daily, thus being able to hit the target in a more sustained manner. Neuroblastoma cells treated with 4SC‐205 (Figure 2E; Table S5) displayed all the expected phenotypic features resulting from KIF11 inhibition such as the inability to form bipolar spindles (Figures 2F and S4A), cell cycle arrest during mitosis (Figure S4B‐H), and induction of apoptosis (Figure S5), thereby confirming the high KIF11 specificity of this compound. While similar effects were observed in 3D spheroid cultures (Figure S6A‐C), 4SC‐205 did not affect the viability of differentiated cells (Figure S6D‐G).

**FIGURE 2:**
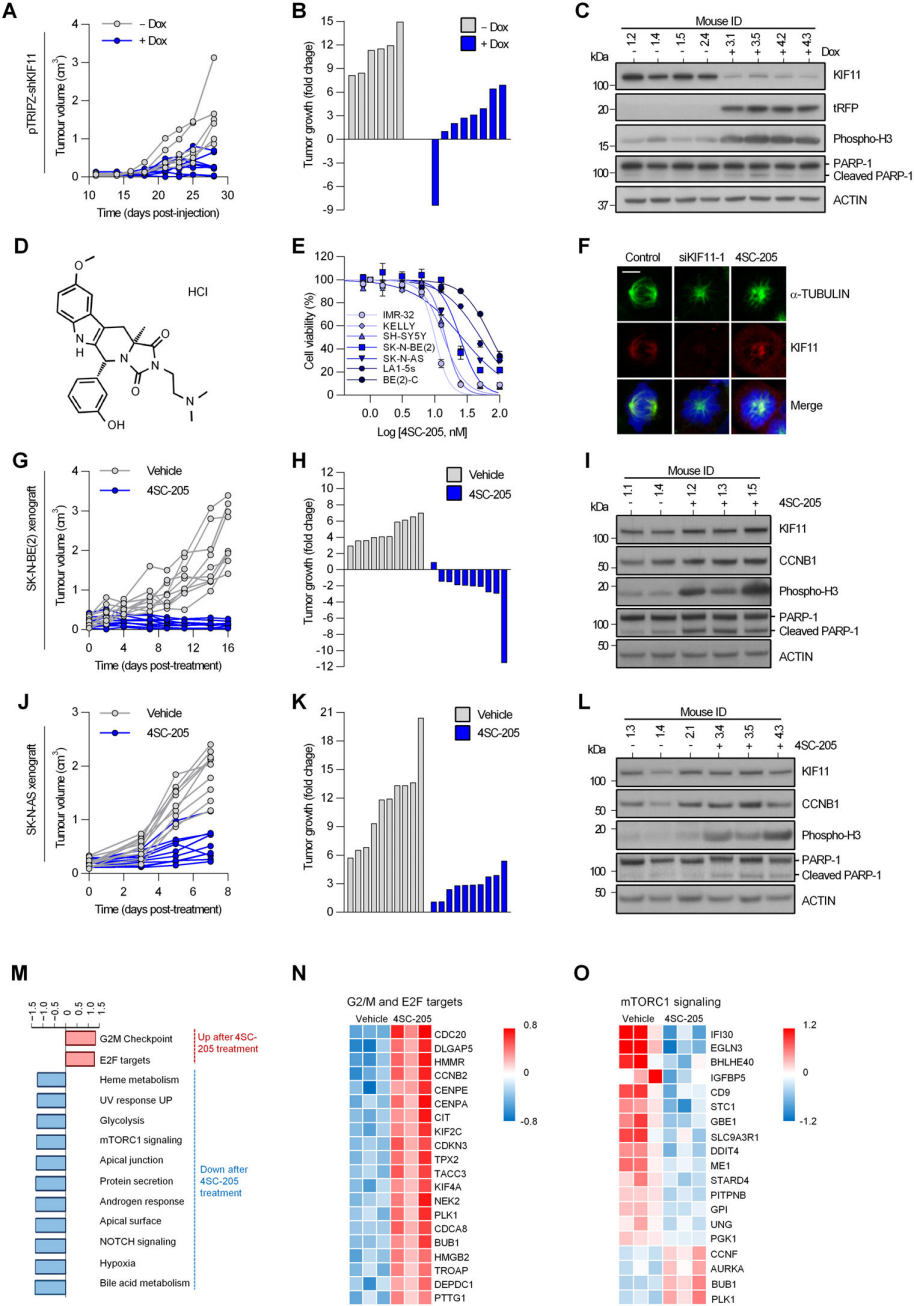
Genetic and pharmacological inhibition of KIF11 reduces tumor growth in subcutaneous neuroblastoma xenografts. (A) Analysis of tumor volume of SK‐N‐BE(2) cells transduced with an inducible shKIF11 lentiviral construct comparing the effects of KIF11 silencing (+Dox) versus control (‐Dox). (B) Waterfall plot comparing the change in tumor volume at day 28 post‐injection versus day 18, when doxycycline was added into the drinking water. (C) Western blot analysis of excised tumors at termination of the experiment. Turbo‐RFP (tRFP) reporter expression was used as a control for shRNA transgene induction. (D) 4SC‐205 chemical structure. (E) Dose‐response curves of neuroblastoma cell lines treated with increasing concentrations of 4SC‐205 for 48 h. IC_50_ values are represented as the average of three independent experiments ± SEM. (F) Mitotic spindle immunofluorescence of SK‐N‐BE(2) cells transfected with siKIF11 or treated with 4SC‐205 (25 nM) for 24 h. KIF11: red, α‐TUBULIN: green, DAPI: blue. Scale bar, 5 μm. (G) Individual tumor growth of xenografts derived from SK‐N‐BE(2) comparing vehicle (*n* = 10) versus 40 mg/kg 4SC‐205 (n = 10). (H) Waterfall plot comparing the change in tumor volume at day 16 post‐treatment versus day 4. (I) Western blot analysis of cell‐cycle and apoptosis‐related proteins in SK‐N‐BE(2) resected tumors. (J) Tumor growth of subcutaneous xenograft derived from SK‐N‐AS treated with vehicle (n = 10) or 40 mg/kg 4SC‐205 (*n* = 10). (K) Tumor volume fold change at day 7 post‐treatment versus day 0. (L) Western blot analysis of resected tumors at the end of the experiment. (M) Gene set enrichment analysis of SK‐N‐BE(2) xenografts treated with vehicle or 4SC‐205. Graph represents normalized enrichment score (NES) values of enriched sets with *p* < 0.05. (N and O) Heatmap representing top 20 differentially deregulated genes of G2/M checkpoint and E2F targets (N), and mTORC signaling‐related genes (O)

When used in vivo, 4SC‐205 treated mice showed a remarkable shrinkage of the original SK‐N‐BE(2) subcutaneous xenograft (Figures 2G and 2H) or tumor growth delay in SK‐N‐AS xenografts (Figures 2J, 2K, and Figure S7). Increased phosphorylation of histone H3 and apoptotic hallmarks (i.e., processing of PARP) confirmed that the antitumor effect of 4SC‐205 was comparable to that of genetic KIF11 silencing in vivo (Figures 2I,L). Transcriptomic analysis of the 4SC‐205‐treated tumors confirmed the expected genetic changes of arrested cells in mitosis and tumor cells with reduced proliferation or viability (Figure 2M–O). We next tested 4SC‐205 in a patient‐derived orthotopic xenografts (PDOX) derived from a very high‐risk neuroblastoma patient. VH‐NB608 PDOX retained most of the histological and molecular features of the original tumor (Figure 3A–D). 4SC‐205‐treated mice displayed a 14.75‐fold reduction in tumor weight compared to the vehicle group (Figures 3E and 3F). Mice treated with 4SC‐205 presented small tumors located in the adrenal gland, whereas vehicle‐treated mice had large tumors with the kidney completely surrounded by the tumor (Figure 3G). Furthermore, 4SC‐205 tumors had a larger fraction of cells with phosphorylation of histone H3, thereby indicating a specific targeting of KIF11 in these tumors, and suggesting that tumors were still sensitive to the inhibitor after 3 weeks (Figure 3H,I).

**FIGURE 3:**
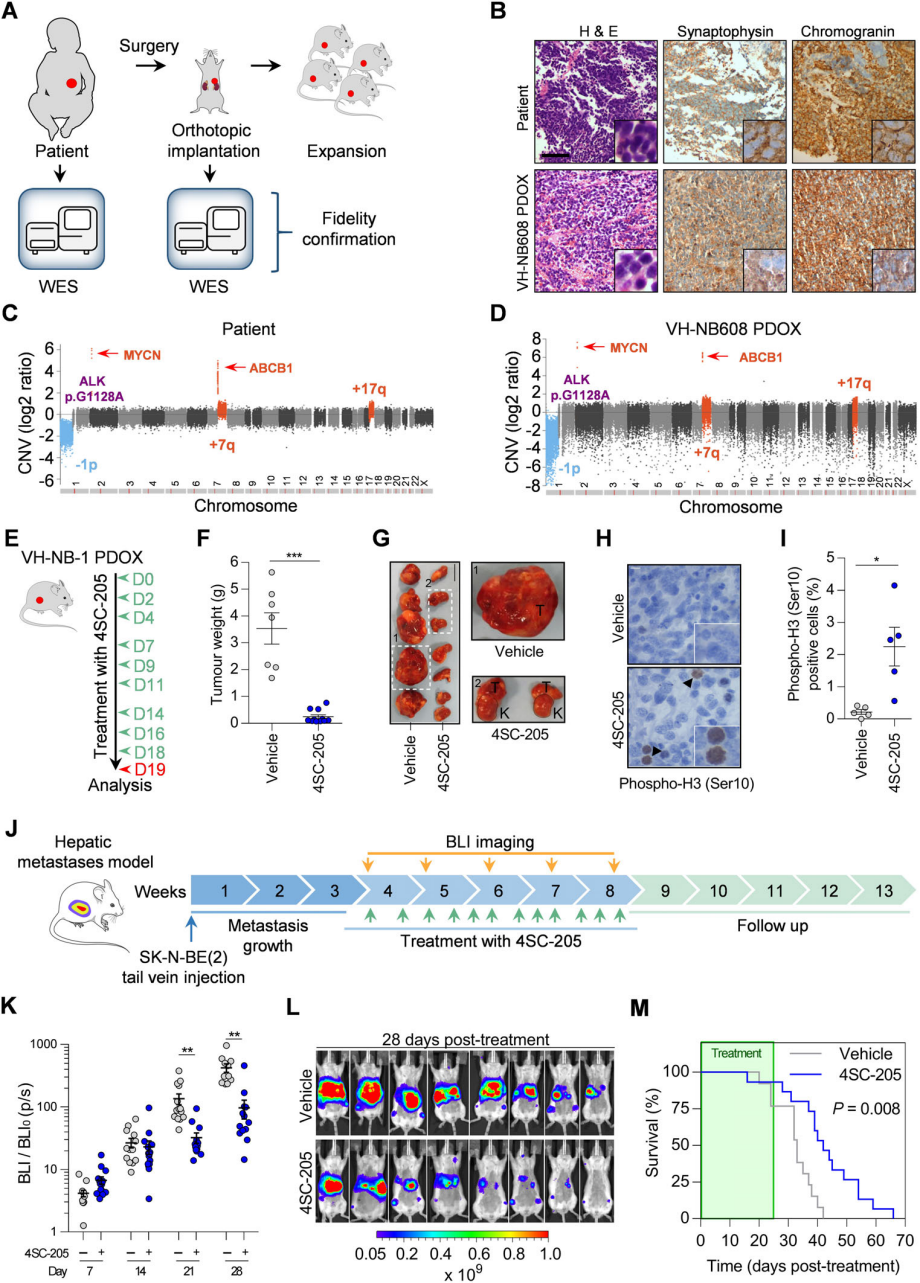
4SC‐205 impairs PDOX growth and prolongs the survival of mice bearing neuroblastoma liver metastasis. (A) Schematic representation of PDOX generation and characterization. (B) Immunohistochemistry of neuroblastoma markers in FFPE tumor sections from the original tumor (upper panels) and after implantation in mice (lower panels). H&E: Hematoxylin and eosin staining. Scale bar represents 110 μm. (C and D) Chromosomal copy number variations (CNV) of the original tumor and after implantation in mice. The most relevant molecular pathogenic alterations found in the original tumor and PDOX are highlighted. (E) Schematic illustration of the treatment schedule. Mice bearing PDOX were treated for 3 weeks with either vehicle (*n* = 7) or 4SC‐205 (40 mg/kg, *n* = 11). (F) Tumor weights at the end of the experiment. (G) Representative picture of the dissected tumors (T: tumor; K: kidney). Scale bar: 1 cm. (H) Representative images of phosphorylated histone H3. Scale bar: 10 μm. (I). Quantification of phospho‐histone H3 positive cells in histological sections from vehicle‐ and 4SC‐205‐treated tumors. Graph represents the average percentage of positive cells ± SEM from vehicle‐or 4SC‐205 (10 representative fields/tumor) ‐treated tumors (*n* = 5/group). (J) Scheme of the experimental design. SK‐N‐BE(2) cells were injected into the tail vein, and 21 days later, mice were randomized into vehicle (*n* = 13) and 40 mg/kg 4SC‐205 groups (*n* = 15). The mice received oral administration of 4SC‐205 three times a week for five consecutive weeks. (K) Scatter dot plots representing the average quantification of tumor bioluminescence ± SEM at the indicated days post‐treatment. **p < 0.01, two‐tailed student's *t*‐test. (L) Representative images of luciferase activity in eight mice from the vehicle and 4SC‐205 treatment groups at 28 days posttreatment. (M) Kaplan–Meier survival curve of mice with neuroblastoma liver metastases treated with either vehicle or 40 mg/kg 4SC‐205 for 5 weeks. Statistical differences were calculated using the Gehan–Breslow–Wilcoxon test. *p < 0.05, ***p < 0.001, two‐tailed Student's *t*‐test

Half of neuroblastoma patients present metastases at the time of diagnosis.^8^ Therefore, we proceeded to test the efficacy of 4SC‐205 in a neuroblastoma liver metastasis model. In response to treatment, a clear delay in metastatic outgrowth was observed in 4SC‐205‐treated mice (Figure 3J–L and S8). As a consequence, the median lifespan of the animals was significantly expanded by ∼27% (Figure 3M; vehicle: 33 days *vs*. 4SC‐205: 42 days). Noticeable, 4SC‐205 administration minimally affected mice weight (<10%) during the course of the treatment (Figure S9). To achieve a better therapeutic effect and provide a rationale for further development of 4SC‐205 in clinical trials, we combined 4SC‐205 with chemotherapies, such as platine derivatives (cisplatin), doxorubicin, and topotecan, which are currently used as standard treatment for patients with high‐risk neuroblastoma. In all cases, the combination of 4SC‐205 with the chemotherapies showed additive effects (Figures 4A–D and S10A‐C; Table S6). Pediatric precision medicine programs have discovered a small number of recurrent alterations such as *ALK* activating mutations or hyperactivation of the ERK Pathway,^9^, ^10^ which constitute the basis for the development of targeted therapies against high‐risk neuroblastoma tumors. Thus, we combined 4SC‐205 with two ALK inhibitors (ceritinib and lorlatinib) or with the MEK1/2 inhibitor selumetinib. The combination of 4SC‐205 with ALK or MEK inhibitors showed a ∼2–3‐fold reduction in cell proliferation compared with the inhibitors alone, with most of the combination doses showing additive effects (Figures 4E–J and S10D‐G; Table S7).

**FIGURE 4:**
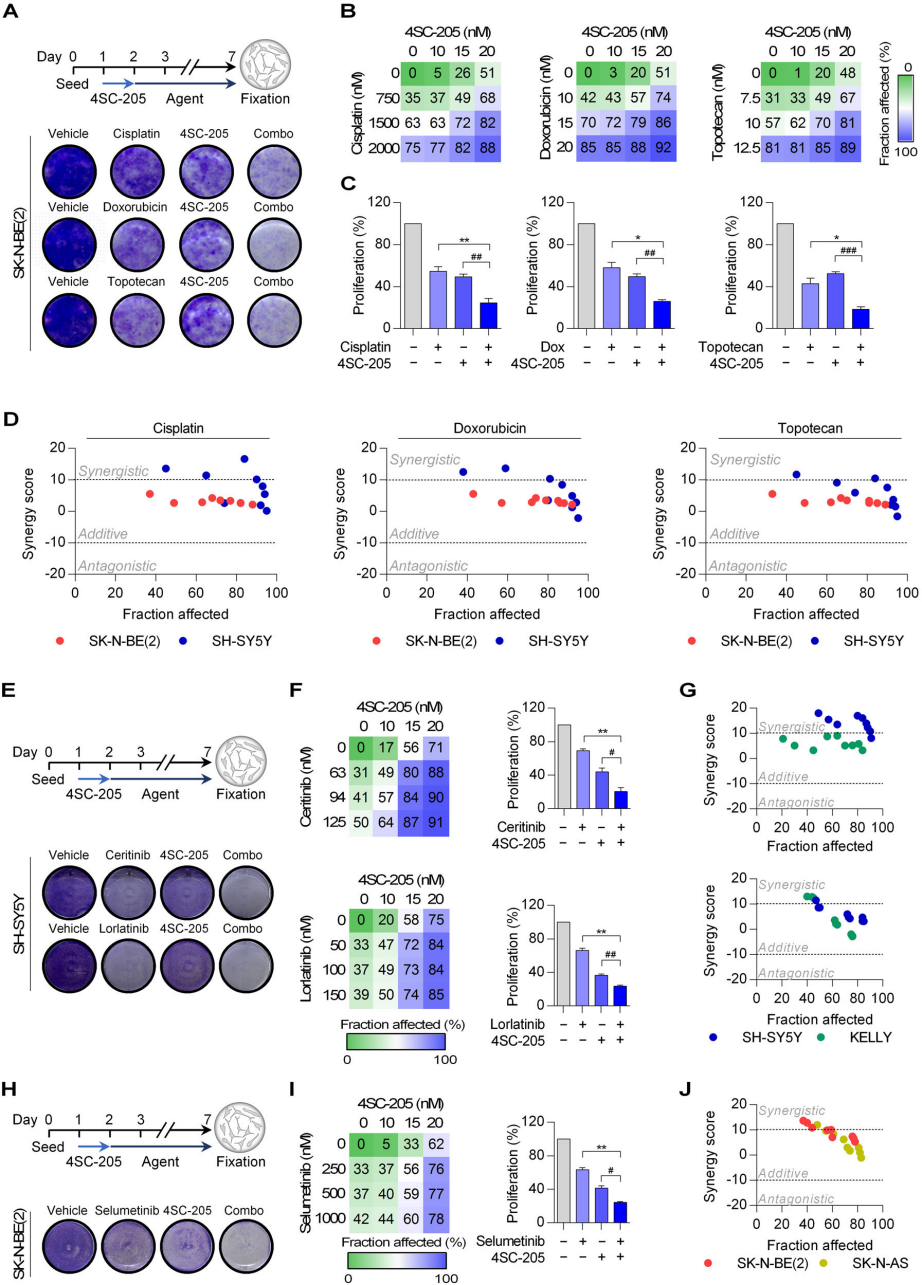
4SC‐205 potentiates the effect of chemotherapy and neuroblastoma‐targeted therapies. (A) Scheme of the experimental design for the combination of 4SC‐205 and standard chemotherapies. Images are representative of crystal violet staining of SK‐N‐BE(2) cells treated with CDDP (1000 nM), doxorubicin (15 nM), topotecan (10 nM), 4SC‐205 (17.5 nM) and their corresponding combinations (Combo). (B) Heatmaps showing the percentage of cellular fraction affected by drug combination treatments. (C) Graphs represent the average effect on cell viability from three independent experiments ± SEM (*n* = 3/condition). (D) Combinatorial analysis performed using SynergyFinder 2.0 software. (E) Schematic representation of the experimental design combining 4SC‐205 and neuroblastoma‐targeted therapies. Representative crystal violet staining images of the ALK‐mutated SH‐SY5Y cell line treated with the ALK inhibitors ceritinib (62.5 nM) or lorlatinib (50 nM) with or without 4SC‐205 (15 nM). (F) Heatmaps show the percentage of the cellular fraction affected by the drug combination treatments at the indicated doses. The graph represents the average of three independent experiments (*n* = 3/condition) ± SEM at 62.5 nM and 50 nM of ceritinib or lorlatinib, respectively. (G) Combinatorial analysis of 4SC‐205 and ceritinib/lorlatinib in SH‐SY5Y and KELLY cells. (H) Crystal violet staining images of SK‐N‐BE(2) cells treated with selumetinib (250 nM) and 4SC‐205 (20 nM) and their respective combinations. (I) Heatmap showing the fraction of cells affected after the combination of 4SC‐205 and selumetinib at the indicated concentrations for 48 h. Graph represents the average percentage of cell proliferation (*n* = 3/condition) ± SEM at 250 nM of selumetinib. (J) Combinatorial analysis of 4SC‐205 and selumetinib in SK‐N‐BE(2) and SK‐N‐AS. *p < 0.05, **p < 0.01, ***p < 0.001, two tailed Student's *t*‐test

In summary, our study provides a rationale for the future therapeutic integration in clinical trials of 4SC‐205, an structurally distinct oral KIF11 inhibitor that shows potent antitumor activity in multiple preclinical neuroblastoma models and sensitizes neuroblastoma cells to standard chemotherapy and specific neuroblastoma‐targeted therapies.

## CONFLICT OF INTEREST

Dr. Moreno participates in data monitoring committees of clinical trials sponsored by Novartis, Actuate Therapeutics, Shionogi, Incyte, the University of Southampton and the Royal Marsden NHS Foundation Trust; and had a consulting role for Novartis and Shionogi. Dr. Lucas Moreno is also a member of the Executive Committee of the European neuroblastoma research cooperative group (SIOPEN) which receives royalties for the sales of dinutuximab beta. Rolf Krauss (RK) is an employee of 4SC. Alberto Villanueva (AV) is co‐founder of Xenopat S.L. No potential conflict of interest was disclosed by the rest of the authors.

## AUTHOR CONTRIBUTIONS

Miguel F. Segura and Anna Santamaria conceived and designed the study. Marc Masanas, Nuria Masiá, Leticia Suárez‐Cabrera, Mireia Olivan, Aroa Soriano, Blanca Majem, Carlos Jimenez, Ariadna Boloix, Ignasi Toloedano, Gabriela Guillén, Alexandra Navarro, and Alberto Villanueva carried out the experiments. Marc Masanas, Laura Devis‐Jauregui, Rebeca Burgos‐Panadero, Pau Rodriguez‐Sodupe, and Aroa soriano analyzed the data. David Llobet‐Navas, Josep Sánchez de Toledo, Josep Roma, Rosa Noguera, Lucas Moreno, and Soledad Gallego provided intellectual support for result interpretation and critical revision. Rolf Krauss provided the compound used in this study. Marc Masanas, Miguel F. Segura, and Anna Santamaria wrote the initial manuscript. All the authors read and approved the final manuscript.

## Supporting information

Supporting Information

## Data Availability

The authors confirm that all data supporting the findings of this study are available within the article and the corresponding web servers. Further information from WES data analyses is available from the corresponding authors upon reasonable request.
