# Supplementary material for: The oral KIF11 inhibitor 4SC‐205 exhibits antitumor activity and potentiates standard and targeted therapies in primary and metastatic neuroblastoma models
Source: Clin Transl Med. 2021 Oct 14;11(10):e533. doi: 10.1002/ctm2.533 (PMC8516339; doi:10.1002/ctm2.533)
Supplement: Supplementary file 1 — Supporting Information [file CTM2-11-e533-s001.docx]

**The oral KIF11 inhibitor 4SC-205 exhibits antitumor activity and potentiates standard and targeted therapies in primary and metastatic neuroblastoma models**

Marc Masanas et al.

**SUPPORTING INFORMATION**

**Materials and methods**

**Analysis of mRNA neuroblastoma data sets**

*KIF11* mRNA expression was analyzed with the R2 software: Genomics Analysis and Visualization Platform (http://r2.amc.nl) using SEQC498 (GSE62564), Kocak (GSE45547), and Maris (GSE3960) datasets. Receiver operating characteristic curves were constructed to determine the diagnostic power of KIF11 expression for clinical outcome prediction using the SEQC498 dataset. The optimal cutoff value was defined according to the Youden index. *KIF11* expression was categorized as “high” (≥ median) and “low” (<median). Overall survival (OS) and the cumulative survival rate were estimated using the Kaplan-Meier method, and the log-rank test was performed to assess differences between groups. Univariate and multivariate Cox proportional hazard regression analyses were used to assess the prognostic significance of *KIF11* on survival. The Maris (GSE3960) dataset was used to correlate *KIF11* mRNA expression with *MYCN* amplification, gain of 17q23, loss of heterozygosity of 1p36 and 11q genomic alterations. All statistical analyses were performed using the IBM SPSS 21. All reported *p* values were based on two-sided tests with *p* values <0.05, which were considered statistically significant.

**Immunohistochemistry**

Twenty-five primary non-*MYCN*-amplified neuroblastoma samples (at least two representative cylinders of 1 mm^2^ from each tumor) classified according to the International Neuroblastoma Risk Group (INRG) pre-treatment stratification criteria ([1](#_ENREF_1)) were included in five tissue microarrays. Patient samples were referred to the Spanish Reference Center for Neuroblastoma Biological and Pathological studies (Department of Pathology, University of Valencia-INCLIVA) between 2008 and 2010. All patients, their relatives, or their legal guardians provided written informed consent. The present study was approved by the Clinical Research Ethics Committee of INCLIVA (ref. B.0000339).

For immunostaining, tissue sections were deparaffinized overnight at 60°C and rehydrated using graded alcohols. Heat-induced antigen retrieval was performed using citrate buffer (pH 6, 4 min, 115°C) in a pressurized heating chamber. Primary antibodies (Table S8) were incubated overnight at 4°C after blocking endogenous peroxidase. Tissue sections were incubated with secondary antibody for 30 min at room temperature (RT), developed using diaminobenzidine (Dako, K3468), and counterstained using hematoxylin. The assessment of immunostained sections was performed by two independent studies according to the intensity and percentage of KIF11-positive tumor cells. The intensity of positive cells was scored as 1 (weak), 2 (moderate), or 3 (strong), and the percentage of cells as 1 (1–25%), 2 (>25–50%), 3 (>50–75%), or 4 (>75–100%), as per previous studies ([2](#_ENREF_2)). The sum of these parameters allowed us to categorize the samples as follows: a staining score of ≤ 3 was considered as low expression, and a score of ≥3 was considered as high expression. The association between KIF11 protein expression and INRG features was analyzed using the χ² test. Survival analysis was performed using Kaplan-Meier curves and log-rank tests. Statistical significance was set at *p* < 0.05. All data were analyzed using SPSS 26.0 statistical analysis software (SPSS, Inc., Chicago, IL, USA).

**Cell lines**

SK-N-AS, SH-SY5Y, IMR-32, SK-N-F1, SK-N-BE(2)-C, and HEK293T cell lines were purchased from the American Type Culture Collection. SK-N-BE(2), KELLY, and LA1-5s were purchased from Public Health England Culture Collection. The CHLA-90 cell line was acquired from the Children’s Oncology Group Cell Culture and Xenograft Repository. NBL-S and NGP were provided by the Cell Bank DSMZ (German Collection of Microorganisms and Cell Cultures). All cell lines were amplified and stored in liquid nitrogen. Upon resuscitation, the cells were maintained in culture for no more than two months. Cell lines were cultured and maintained in Iscove’s Modified Dulbecco’s medium (Thermo Fisher Scientific) supplemented with 1% insulin-transferrin-selenium supplement (Thermo Fisher Scientific). KELLY cells were cultured in RPMI 1640 medium (Thermo Fisher Scientific). HEK293T cells were grown in Dulbecco’s modified Eagle medium (Thermo Fisher Scientific). All media were supplemented with 10% heat-inactivated fetal bovine serum (South America Premium, Biowest), 100 U/mL penicillin, 100 µg/mL streptomycin (Thermo Fisher Scientific), and 5 μg/mL plasmocin (InvivoGen). All cultures were maintained at 37°C in a humidified atmosphere of 95% air and 5% CO_2_ and periodically tested for mycoplasma contamination.

**Inducible MYCN silencing**

The MYCN-Tet-off-inducible Tet21N cell line was a generous gift from Manfred Schwab (DKFZ, Heidelberg, Germany). Cells were cultured in RPMI 1640 medium (Thermo Fisher Scientific) supplemented with 10% heat-inactivated fetal bovine serum (South America Premium, Biowest), 25 mM HEPES, 4 mM L-Glutamine, 200 ug/mL G418, 0.5 ug/mL amphotericin B, 10 ug/mL hygromycin B, 100 U/mL penicillin, 100 ug/mL streptomycin (Thermo Fisher Scientific), and 5 μg/mL plasmocin (InvivoGen). MYCN depletion was triggered by the addition of 100 ng/mL doxycycline.

**MYCN overexpression**

pCDNA3-HA-human MYCN was a gift from Martine Roussel (Addgene plasmid # 74163; http://n2t.net/addgene:74163; RRID:Addgene_74163)([3](#_ENREF_3)). For MYCN overexpression 2.5 x 10^3^ SK-N-AS or SH-SY5Y were seeded in 35 mm dishes and reverse transfected using Lipofectamine 2000 (Thermo Fisher Scientific; 3 µL/plate) with increasing concentrations of pCDNA3-HA-human MYCN or empty vector (0, 1, 2 or 4 µg/p35). Forty-eight h later, cells were harvested and protein was extracted for western blot analyses.

**Quantitative real-time PCR**

Total RNA was isolated using the miRNeasy Mini Kit (Qiagen). MRNA (1 µg) were reverse transcribed using a Taqman RT kit (Applied Biosystems, Thermo Fisher Scientific). Real-time PCR of KIF11 and MYCN was performed using 2X Power SYBR Green Master Mix (Applied Biosystems, Thermo Fisher Scientific). MRNA expression was normalized against the *GAPDH* housekeeping gene. Primer sequences are listed in Table S9. Relative quantification of gene expression was performed with a comparative 2^(-ΔΔCT)^ method ([4](#_ENREF_4)).

**Lentivirus production, transduction and isolation of clones**

Lentiviruses containing pTRIPZ (Dharmacon, GE Healthcare) or pTRIPZ-shKIF11 (Dharmacon, GE Healthcare; V3THS_391757) were produced using previously described methods in HEK293T ([5](#_ENREF_5)). Virus particles were concentrated by ultracentrifugation. SK-N-BE(2) were seeded (3 × 10^5^ cells/dish) in 60-mm dishes and incubated overnight with lentiviruses. Infected cells were selected with 1 µg/mL puromycin (Sigma-Aldrich). To isolate monoclonal population of pTRIPZ-KIF11, transduced SK-N-BE(2) were seeded at very low density into 100-mm dishes. After 10 days, isolated colonies were sorted and amplified in 96-well plates.

***In vivo* experiments**

*Subcutaneous xenografts:* SK-N-BE(2) and SK-N-AS were subcutaneously injected into the right flank (5 × 10^6^ cells/flank) of 6-week old female Fox Chase SCID mice (Charles River) in 300 µL of PBS: Matrigel (1:1). When tumor size reached 100–200 mm^3^, mice were randomized into control (2% sucrose) and shKIF11. KIF11 knockdown was induced by adding 1 mg/mL doxycycline to drinking water.

For drug efficacy experiments, mice bearing measurable tumors (~100–200 mm^3^) were randomized into two groups and treated three times per week by oral gavage with vehicle (5% polyethylene glycol 400 (PEG400, Fisher Scientific, 11449467), 0.9% NaCl, pH 3–4) or with 40 mg/kg 4SC-205. Tumor volume was measured every 2–3 days. At the end of the experiment, tumors were dissected, weighed, frozen in liquid nitrogen, fixed in 10% formalin, and embedded in paraffin.

*Orthoxenografts or patient-derived orthotopic xenograft model (PDOX)*: A biopsy of a primary tumor located in left adrenal gland of a 7-month old female patient with high risk metastatic neuroblastoma was performed at diagnosis at the Vall d’Hebron University Hospital (VH), Barcelona, Spain. The specimen was aseptically isolated and placed at RT in IMDM (Life Technologies, Thermo Fisher Scientific) supplemented with 20% FBS (South America Premium, Biowest), 1% of insulin–transferrin–selenium supplement plus 100 U/ml penicillin and 100 μg /ml streptomycin (Life Technologies, Thermo Fisher Scientific) and 5 μg/mL plasmocin (InvivoGen). The protocol for the use of patient’s tumor sample was reviewed and approved according to Ethical Committee of Clinical Research and written informed consent was collected.

Immediately after biopsy the trucut was implanted into a NU-Foxn1nu mouse (Harlan) at the animal core facility of Bellvitge Biomedical Research Institute (IDIBELL). A seven weeks-old female mouse was anesthetized with a continuous flow of 2% to isoflurane/oxygen mixture; the sample was implanted without enzymatic digestion in the left adrenal gland of the mice using a 7.0 suture and monitored twice weekly measuring their body weight and by palpation of tumors. At 177 days post-implantation, the tumor was extracted, cut into small fragments and serially implanted into 3 to 5 new animals. Engrafted tumors were also cryopreserved in a solution of 90% non-inactivated FBS and 10% dimethyl-sulfoxide, stored in liquid nitrogen for subsequent future implantations, and frozen for advanced molecular analysis. Representative tumor fragments were fixed and then processed for paraffin embedding.

For drug efficacy experiments, eighteen nude mice were implanted with PDOX at passage two. Sixty-five days after implantation, when tumors measured 300–400 mm^3^, mice were randomized and assigned to the vehicle and 4SC-205 groups. At the end of the experiment, tumors were dissected, weighed, frozen in liquid nitrogen, fixed in 10% formalin, and embedded in paraffin.

*Neuroblastoma liver metastasis model*: Firefly luciferase-transduced SK-N-BE(2) cells were injected into the lateral tail vein (2.5 × 10^5^ cells/mouse in 150 µL of PBS) of 5–6-week-old female Fox Chase SCID Beige mice (Charles River). Twenty-four days after injection, the mice were randomized into vehicle and 4SC-205 groups. Metastasis growth was monitored by *in vivo* bioluminescence imaging (IVIS) once a week during 5 weeks. All animal protocols were reviewed and approved according to regional Institutional Animal Care and Ethical Committee of Animal Experimentation.

**Immunofluorescence**

SK-N-BE(2) and SH-SY5Y cells were seeded in 6-well plates (2 × 10^5^ cells/well) on 15 mm coverslips and left for 24 h before drug treatment. Cells were seeded in 6-well plates (7.5 × 10^5^ cells/well), and for siRNA experiments, reverse transfected with control or KIF11 siRNA oligonucleotides (25 nM) (Table S10) using Lipofectamine 2000 (Thermo Fisher Scientific; 5 µL/well). Alternatively, 24 h after seeding, cells were treated using 25 nM 4SC-205. At the end of the experiment, cells were fixed using PTEMF buffer (50 mM Pipes, 0.2% Triton X-100, 10 mM EGTA, 1 mM MgCl_2_, 4% formaldehyde) for 10 min and washed twice with PBS. The samples were then blocked with 3% BSA in PBS for 30 min at RT. Primary antibodies (Table S8) were diluted in 3% BSA in PBS and incubated for 1 h at RT. Secondary antibodies, DAPI and FITC-α-tubulin, were incubated for 1 h at RT in the dark. The coverslips were mounted with a mounting medium (P36965, Thermo Fisher Scientific).

**Cell cycle analysis**

SK-N-BE(2) (2.5 × 10^6^) and SH-SY5Y (3.5 × 10^6^) cells were seeded in 100-mm dishes. After 14 h, cells were treated with either vehicle (DMSO) or 25 nM 4SC-205. At 12 and 24 h post-treatment, cells were harvested and fixed with cold 70% ethanol and kept at 4°C for at least 24 h. Cells were washed with PBS and resuspended in propidium iodide (PI) buffer (500 µg/mL PI, RNAse 10 µg/mL). DNA content was analyzed using a FACSCalibur flow cytometer (BD Biosciences) and data were analyzed using BD CellQuestTM Pro Software (BD Biosciences).

**Western blot**

Cells were harvested in RIPA buffer 1X (Thermo Fisher Scientific) supplemented with a 1X EDTA-free complete protease inhibitor cocktail (Roche). Tumors were cut into small pieces, disaggregated using a homogenizer (10 s, three times), and sonicated (5 s, three times) in RIPA buffer 1X with 1X EDTA-free complete protease inhibitor cocktail and phosphatase inhibitor cocktail 2 and 3 (Sigma-Aldrich; P5726 and P0044). Protein extracts (20-40 μg of protein) were resolved on NuPAGE 4–12% Bis-Tris gels and transferred to PVDF membranes (Thermo Fisher Scientific). Membranes were blocked with Tris-buffered saline with Tween-20 containing 5% BSA or 5% non-fat dry milk for 1 h at RT and incubated overnight at 4°C with the appropriate primary antibody (Table S8). Membranes were incubated with the corresponding secondary antibodies for 1 h at RT. Actin was used as loading control. Membranes were developed using the EZ-ECL chemiluminescence detection kit (Fisher Scientific).

***In vitro* drug sensitivity assays**

For cell proliferation assays, neuroblastoma cells were seeded in 96-well plates (3.5–14 × 10^3^ cells/well) and treated 24 h later with vehicle (DMSO) or the indicated drugs. Cells were then fixed with 1% glutaraldehyde (Sigma-Aldrich) and stained with 0.5% crystal violet (Sigma-Aldrich). Crystals were dissolved in 15% acetic acid (Fisher Scientific) and the optical density was read at 590 nm using an Epoch microplate spectrophotometer (Biotek). Dose-response curves were calculated using nonlinear regression approximation in GraphPad Prism 6.0 (GraphPad Software, Inc.).

**Cell death assay**

For gene silencing experiments, SK-N-BE(2) and SH-SY5Y cells were seeded in 6-well plates (3 × 10^5^ cells/well) in triplicate and reverse transfected with control or KIF11 siRNA (25 nM) using Lipofectamine 2000 (Thermo Fisher Scientific, 5 µL/well). For drug-induced cell death, cells were seeded in 6-well plates (2 × 10^5^ cells/well) and treated 24 h later with DMSO or 25 nM 4SC-205. At the indicated times, cells were stained using 0.05 µg/mL Hoechst 33258 dye and photographed. Apoptosis quantification was performed using eight representative images per well (n = 3 replicates per condition). Cells with fragmented or condensed chromatin were scored as apoptotic cells, whereas uniformly stained chromatin cells were considered healthy.

**Three-dimensional spheroid culture**

For tumor spheroids, SH-SY5Y (4.5 × 10^4^ cells/well) and SK-N-BE(2) (6 × 10^4^ cells/well) cells were seeded in non-adherent 6-well plates and let them grow in spheroids for 48-72 h. Spheroids were then treated with DMSO or 25 nM 4SC-205. Forty-eight h later, spheroids were disaggregated with 0.5 mL of 1X StemPro®Accutase® (Thermo Fisher Scientific) and incubated with a mixture of PMS:MTS (1:20) for 2-5 h. Optical density was read at 590 nm using an Epoch Microplate Spectrophotometer (Biotek).

**Differentiation of neuroblastoma cells**

The protocol followed was adapted from ([6](#_ENREF_6)). 1 × 10^4^ SH-SY5Y/cm^2^ were seeded in 12-well plates and 60-mm dishes pre-coated with poly-D-lysine (Fisher Scientific) and collagen (Fisher Scientific). After 24 h, cells were treated with all-trans retinoic acid (ATRA) (Selleckchem) 10 µM in IMDM 10% FBS without antibiotics for 5 days. Cells were then washed with IMDM without FBS and incubated with fresh media 0.5% FBS and 50 ng/mL brain-derived neurotrophic factor (BDNF) (Sigma-Aldrich). After three days, cells were treated with vehicle (DMSO) or 4SC-205 at 25 nM. Cells were fixed with 1% glutaraldehyde or recollected for western blot analysis at the indicated time points.

**Genomic analyses**

*RNA sequencing*. For transcriptomic analyses, total RNA was isolated from SK-N-BE(2) subcutaneous tumors treated with vehicle or 4SC-205 (n = 3/group) for 24 h using the miRNeasy mini kit (Qiagen). RNA quality and quantity were determined using a Qubit® RNA HS Assay (Life Technologies) and RNA 6000 Nano Assay on a Bioanalyzer 2100 (Agilent). RNASeq libraries were prepared following the TruSeq® Stranded mRNA LT Sample Prep Kit protocol (Illumina) and sequenced on a NovaSeq 6000 system (Illumina). RNAseq reads were mapped against the human reference genome (GRCh38) using STAR software version 2.5.3a ([7](#_ENREF_7)) with ENCODE parameters. Genes were quantified using RSEM version 1.3.0 ([8](#_ENREF_8)) with default parameters and annotation files from GENCODE version 34. Differential expression analysis was performed with the DESeq2 v1.26.0 R package ([9](#_ENREF_9)) using a Wald test to compare the vehicle and treated samples. We considered differentially expressed genes with *p* values adjusted to < 0.05, and absolute fold-change (FC) > 1.5. Functional enrichment analysis of “Hallmarks” gene set collections from MSigDB was performed using GSEA software ([10](#_ENREF_10),[11](#_ENREF_11)).

*Whole exome sequencing (WES)*: Genomic DNA from the patient’s blood, tumor, and PDOX samples were extracted using the DNeasy Blood & Tissue Kit (Qiagen) and quantified using Qubit (Thermo Fisher Scientific). Genomic DNA was fragmented and a sample library was prepared using the KAPA library kit (Roche), hybridized with the SeqCap EZ MedExome capture kit (Roche), and sequenced on a NextSeq500 sequencing system (Illumina) with the 2 x 150 bp paired-end mode. The R-package XenofilteR to remove sequence reads of mouse origin while retaining human sequences (mismatch threshold=8) ([12](#_ENREF_12)) was used.

Copy number variants (CNVs), single nucleotide variants (SNVs), and small insertions/deletions (indels) from somatic samples as well as SNV and indel from germline samples were detected by bioinformatics analysis. The bioinformatics workflow started mapping sequence reads to the human genome build (hg19) using the BWA tool ([13](#_ENREF_13)). Variant calling for the identification of SNVs and indels was carried out using the GATK Haplotype Caller tool ([14](#_ENREF_14)) together with FreeBayes and Strelka2 ([15](#_ENREF_15)) for constitutional DNA and via the VarDict tool ([16](#_ENREF_16)), together with MuTect2 ([17](#_ENREF_17)) and Strelka2 and VarScan2 ([18](#_ENREF_18)) for somatic DNA. Both germline and somatic variants were considered when called by at least two callers. To determine the effect of variants, we used the SnpEff annotation ([19](#_ENREF_19)). Control-FREEC was used to investigate genomic CNV and B-alelle frequency ([20](#_ENREF_20)). Variants were filtered following maximum population frequency <1%, cancer genes list, tumor variant allele frequency >10%, germline allele frequency >30%, and variant consequence (missense, frameshift, splicing, and stop). Filtering parameters for amplifications were number copy >4 and deletions number copy <1.5. RNA sequencing is available at the GEO public repository (GSE166984), and WES data analyses will be available upon request.

**Drug combination studies**

For drug combination studies with standard chemotherapies, SK-N-BE(2) (2 × 10^3^ cells/well) and SH-SY5Y (2.5 × 10^3^ cells/well) cells were seeded in 96-well plates. The next day, the cells were treated with the indicated concentrations of 4SC-205. Twenty-four h later, cell medium was replaced with a medium containing the indicated concentrations of cisplatin, topotecan, and doxorubicin, and incubated for additional 5 days.

In combination with ALK inhibitors, SH-SY5Y (8 × 10^3^ cells/well) and KELLY (1 × 10^4^ cells/well) cells were seeded in 96-well plates and treated 24 h later with increasing concentrations of 4SC-205 plus/minus cerlitinib or lorlatinib. For combinations with MEK inhibitors, SK-N-BE(2) and SK-N-AS (6 × 10^3^ cells/well) cells were seeded in 96-well plates and treated 24 h later with the indicated doses of 4SC-205 plus/minus selumetinib. Forty-eight h later, cells were fixed with 1% glutaraldehyde (Sigma-Aldrich) and stained with 0.5% crystal violet (Sigma-Aldrich).

The Bliss independence model from SynergyFinder (version 2.0) was used to evaluate if the pharmacological combination of 4SC-205 with chemotherapeutic agents and targeted therapies was synergistic, additive or antagonistic ([21](#_ENREF_21)).

**Statistical analyses**

Unless otherwise indicated, statistical significance was determined using an unpaired two-tailed Student’s t-test. Half-maximal inhibitory concentration (IC50) was calculated using nonlinear regression approximation in GraphPad Prism 6.0 (GraphPad Software, Inc.). Differences in survival times among mice treated with vehicle or 4SC-205 were analyzed using the Gehan-Breslow-Wilcoxon test (GraphPad Prism 6.0). * indicates *p* < 0.05, ** indicates *p* < 0.01, and *** indicates *p* < 0.001.

**Supplementary references**

1. Cohn SL, Pearson AD, London WB, Monclair T, Ambros PF, Brodeur GM*, et al.* The International Neuroblastoma Risk Group (INRG) classification system: an INRG Task Force report. *Journal of clinical oncology : official journal of the American Society of Clinical Oncology*. 2009; **27**(2): 289-97.

2. Jin Q, Dai Y, Wang Y, Zhang S, Liu G. High kinesin family member 11 expression predicts poor prognosis in patients with clear cell renal cell carcinoma. *Journal of clinical pathology*. 2019; **72**(5): 354-62.

3. Vo BT, Wolf E, Kawauchi D, Gebhardt A, Rehg JE, Finkelstein D*, et al.* The interaction of Myc with Miz1 defines medulloblastoma subgroup identity. *Cancer cell*. 2016; **29**(1): 5-16.

4. Livak KJ, Schmittgen TD. Analysis of relative gene expression data using real-time quantitative PCR and the 2(-Delta Delta C(T)) Method. *Methods*. 2001; **25**(4): 402-8.

5. Zufferey R, Dull T, Mandel RJ, Bukovsky A, Quiroz D, Naldini L*, et al.* Self-inactivating lentivirus vector for safe and efficient in vivo gene delivery. *J Virol*. 1998; **72**(12): 9873-80.

6. Encinas M, Iglesias M, Lui Y, Wang H, Muhaisen A, Ceña V*, et al.* Sequential treatment of SH-SY5Y cells with retinoic acid and brain-derived neurotrophic factor gives rise to fully differentiated, neurotrophic factor-dependent, human neuron-like cells. *J Neurochem*. 2000; **75**(3): 991-1003.

7. Dobin A, Davis CA, Schlesinger F, Drenkow J, Zaleski C, Jha S*, et al.* STAR: ultrafast universal RNA-seq aligner. *Bioinformatics*. 2013; **29**(1): 15-21.

8. Li B, Dewey CN. RSEM: accurate transcript quantification from RNA-Seq data with or without a reference genome. *BMC Bioinformatics*. 2011; **4**(12): 323.

9. Love MI, Huber W, Anders S. Moderated estimation of fold change and dispersion for RNA-seq data with DESeq2. *Genome biology*. 2014; **15**(12): 550.

10. Subramanian A, Tamayo P, Mootha VK, Mukherjee S, Ebert BL, Gillette MA*, et al.* Gene set enrichment analysis: A knowledge-based approach for interpreting genome-wide expression profiles. *Proceedings of the National Academy of Sciences*. 2005; **102**(43): 15545-50.

11. Mootha VK, Lindgren CM, Eriksson KF, Subramanian A, Sihag S, Lehar J*, et al.* PGC-1α-responsive genes involved in oxidative phosphorylation are coordinately downregulated in human diabetes. *Nature genetics*. 2003; **34**(3): 267-73.

12. Kluin RJC, Kemper K, Kuilman T, de Ruiter JR, Iyer V, Forment JV*, et al.* XenofilteR: computational deconvolution of mouse and human reads in tumor xenograft sequence data. *BMC Bioinformatics*. 2018; **19**(1): 366.

13. Li H, Durbin R. Fast and accurate short read alignment with Burrows-Wheeler transform. *Bioinformatics*. 2009; **25**(14): 1754-60.

14. Van der Auwera GA, Carneiro MO, Hartl C, Poplin R, Del Angel G, Levy-Moonshine A*, et al.* From FastQ data to high confidence variant calls: the Genome Analysis Toolkit best practices pipeline. *Current protocols in bioinformatics*. 2013; **43**: 11.0.1-.0.33.

15. Kim S, Scheffler K, Halpern AL, Bekritsky MA, Noh E, Källberg M*, et al.* Strelka2: fast and accurate calling of germline and somatic variants. *Nature Methods*. 2018; **15**(8): 591-4.

16. Lai Z, Markovets A, Ahdesmaki M, Chapman B, Hofmann O, McEwen R*, et al.* VarDict: a novel and versatile variant caller for next-generation sequencing in cancer research. *Nucleic acids research*. 2016; **44**(11): e108.

17. Benjamin D, Sato T, Cibulskis K, Getz G, Stewart C, Lichtenstein L. Calling Somatic SNVs and Indels with Mutect2. *Preprint at bioRxiv https://wwwbiorxivorg/content/101101/861054v1*. 2019.

18. Koboldt DC, Zhang Q, Larson DE, Shen D, McLellan MD, Lin L*, et al.* VarScan 2: somatic mutation and copy number alteration discovery in cancer by exome sequencing. *Genome research*. 2012; **22**(3): 568-76.

19. Cingolani P, Platts A, Wang le L, Coon M, Nguyen T, Wang L*, et al.* A program for annotating and predicting the effects of single nucleotide polymorphisms, SnpEff: SNPs in the genome of Drosophila melanogaster strain w1118; iso-2; iso-3. *Fly*. 2012; **6**(2): 80-92.

20. Boeva V, Popova T, Bleakley K, Chiche P, Cappo J, Schleiermacher G*, et al.* Control-FREEC: a tool for assessing copy number and allelic content using next-generation sequencing data. *Bioinformatics*. 2012; **28**(3): 423-5.

21. Ianevski A, Giri AK, Aittokallio T. SynergyFinder 2.0: visual analytics of multi-drug combination synergies. *Nucleic acids research*. 2020; **48**(W1): W488-w93.

**Supplementary Figures**


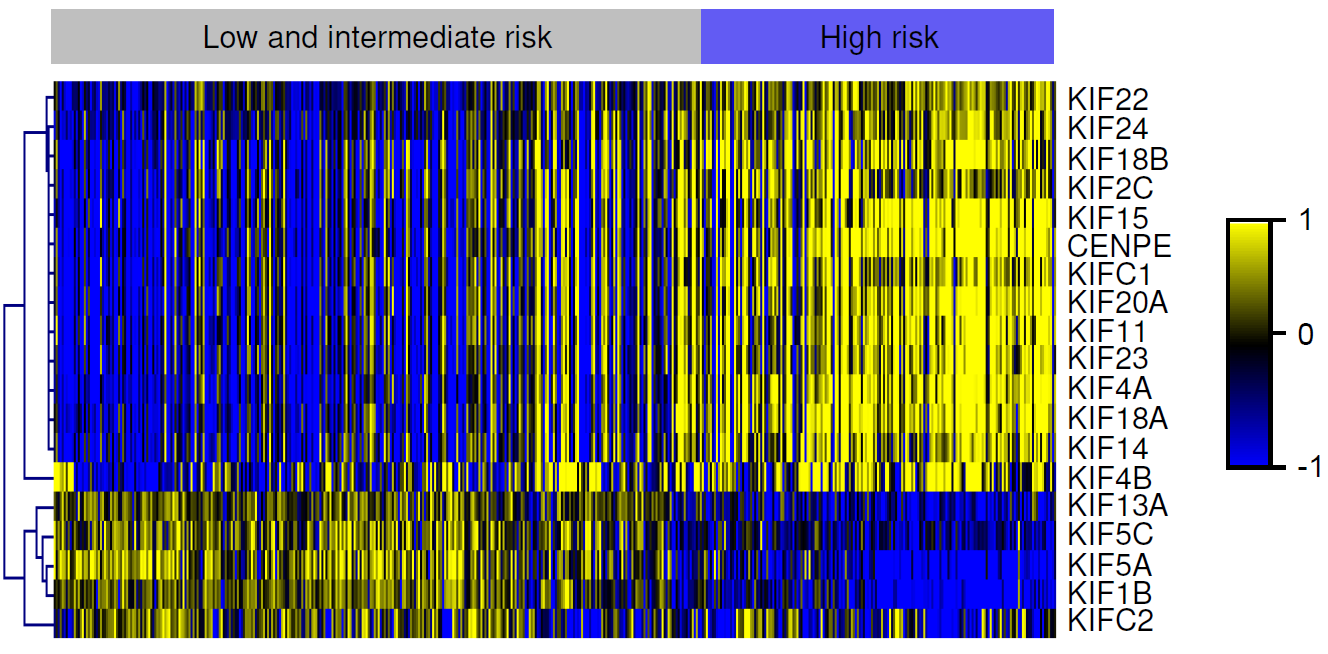


**Figure S1. Multiple kinesins are highly expressed in high-risk neuroblastoma compared to low- and intermediate-risk groups.** Heatmap and hierarchical clustering analysis of the expression of 19 kinesins in 498 NB samples (low- and intermediate-risk, n = 322; high-risk, n = 176) measured by RNAseq.


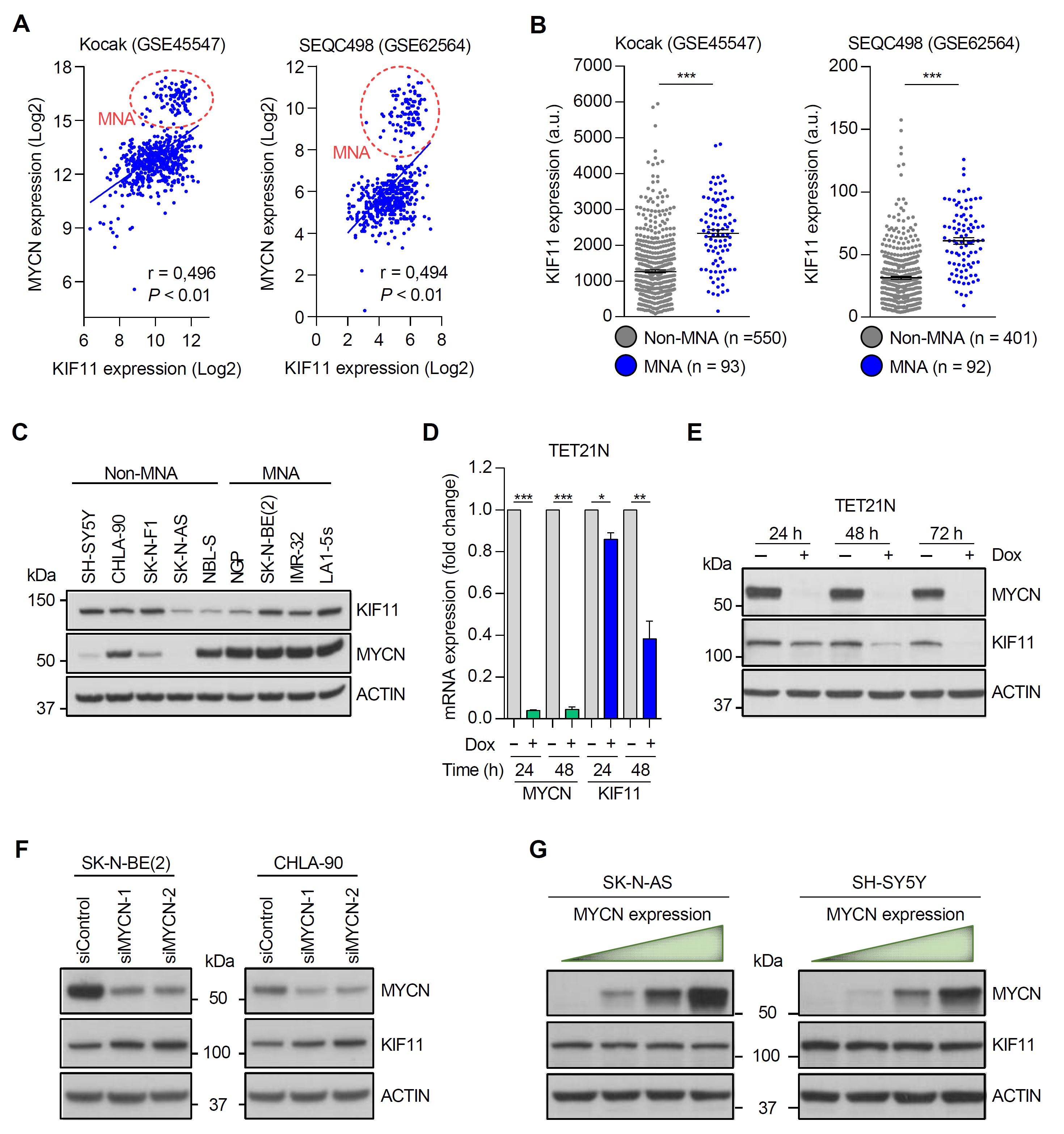


**Figure S2. *KIF11* correlates with *MYCN* in distinct neuroblastoma datasets. (A)** Linear correlation between *MYCN* and *KIF11* mRNA expression in the indicated neuroblastoma datasets. Data from patients with amplification of *MYCN* (MNA) is encircled. (**B**) *KIF11* mRNA expression levels comparing neuroblastoma tumors with amplification of *MYCN* versus no *MYCN* amplification (Non-MNA). (**C)** Western blot analysis of KIF11 in a panel of neuroblastoma cell lines with and without *MYCN* amplification. **(D**) Analysis of *MYCN* and *KIF11* expression in the Tet21N neuroblastoma model with the doxycycline (Dox) inducible silencing of *MYCN*. Results are the mean of three independent experiments ± SEM. * *p* < 0.05, ** *p* < 0.01 and *** *p* < 0.01 two-tailed student’s t-test. (**E**) Western blot analysis of KIF11 in Tet21N cells in the presence of doxycycline (i.e., MYCN depletion) or absence (i.e., MYCN expression). (**F**) Immunoblot analysis of MYCN and KIF11 in SK-N-BE(2) and CHLA-90 cells transfected with two independent siRNAs targeting MYCN 72 h post-transfection. (**G**) KIF11 and MYCN protein expression in SK-N-AS and SH-SY5Y cell lines transfected with increasing concentrations of a MYCN-overexpression vector.


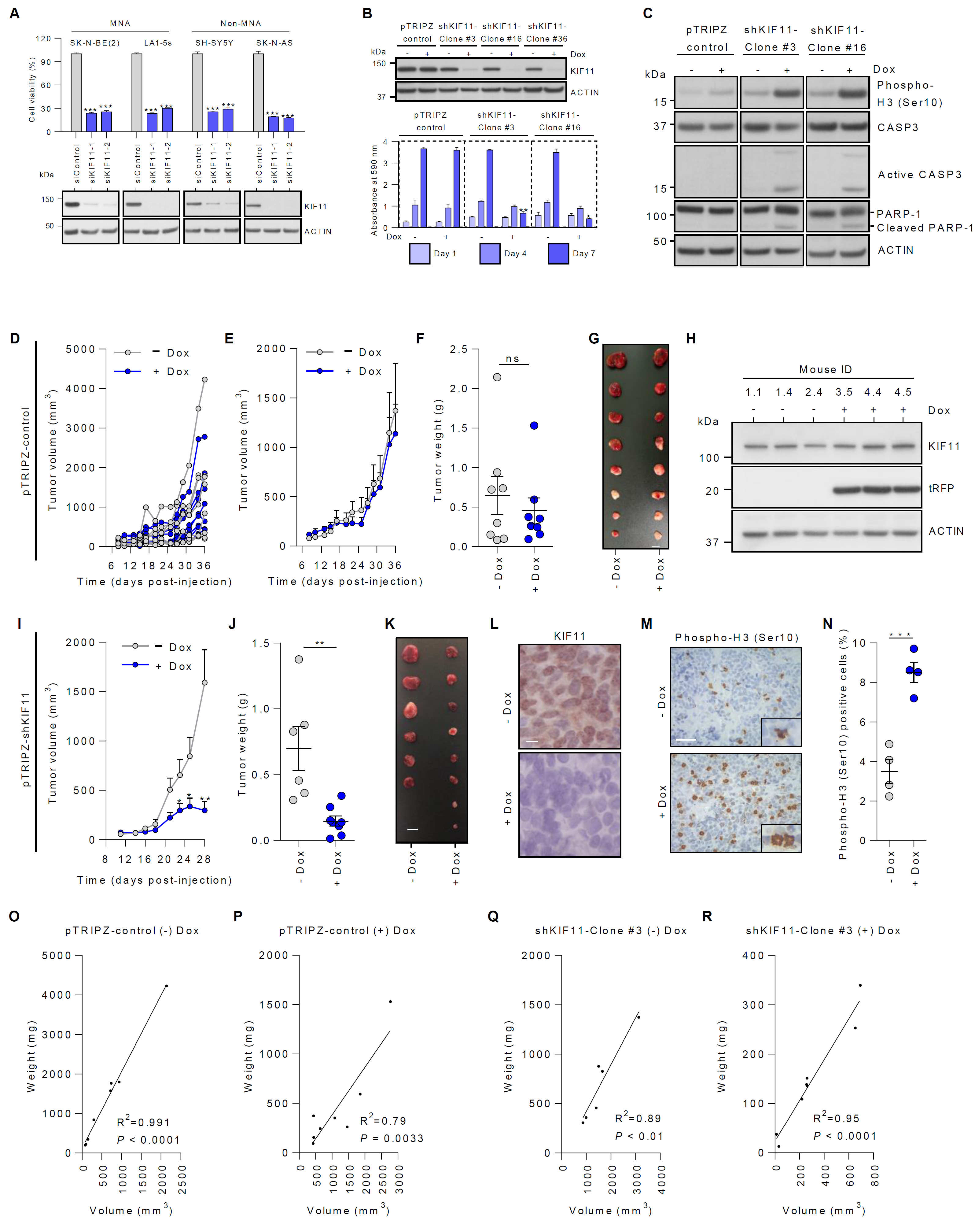


**Figure S3. KIF11 knockdown halts tumor growth and induces apoptosis.** (**A**) Cell viability assay in the indicated cell lines transfected with 25 nM of siControl or siKIF11 at 96 h post-transfection. Protein knock-down was analyzed by western blot 72 h post-transfection (lower panels). (**B**) Western blot analysis of KIF11 in SK-N-BE(2) cells transduced with pTRIPZ-control (pool) or pTRIPZ-shKI11 lentiviral vectors (upper panel). Cells were treated with + / − doxycycline for 96 h. Graph representing cell proliferation after 1, 4 and 7 days upon doxycycline treatment (lower panel). Results are expressed as average of three independent experiments ± SEM. * *p* < 0.05; ** *p* < 0.01; two-tailed Student’s t-test. (**C**) Immunoblot analysis of transduced neuroblastoma cells in presence (+ dox) or absence (− dox) of 1 µg/mL doxycycline for 5 days. (**D, E**) Analysis of individual (**D**) or average (**E**) tumor growth of pTRIPZ-control transduced SK-N-BE(2) xenografts. (**F**) Tumor weight at the end of the experiment. (**G**) Image of the dissected tumors. Scale bar, 1 cm. (**H**) Western blot analysis of resected-tumor samples from pTRIPZ-control. TurboRFP (tRFP) was used as a control of shRNA transgene induction. (**I**) Tumor growth of shKIF11-clone #3 SK-N-BE(2) xenografts comparing control (− dox) *vs* silencing of KIF11 (+ dox). (**J**) Tumor weight at the end of the experiment. (**K**) Image of the dissected tumors. Scale bar, 1 cm. (**L**) Representative image of KIF11 immunohistochemistry in control (− dox) *vs* KIF11 silenced tumors (+ dox). Scale bar, 10 µm. (**M**) Representative image of phospho-histone H3 immunohistochemistry from control (− dox) *vs* KIF11-depleted tumors (+ dox). Scale bar, 100 µm. (**N**) Quantification of phospho-histone H3 positive cells in FFPE tumor sections (n = 4/condition). *** *p* < 0.001, two-tailed Student’s t-test. (**O-R**) Correlation between tumor weight and tumor volume measurements in pTRIPZ-control and shKIF11-Clone #3 xenografts.


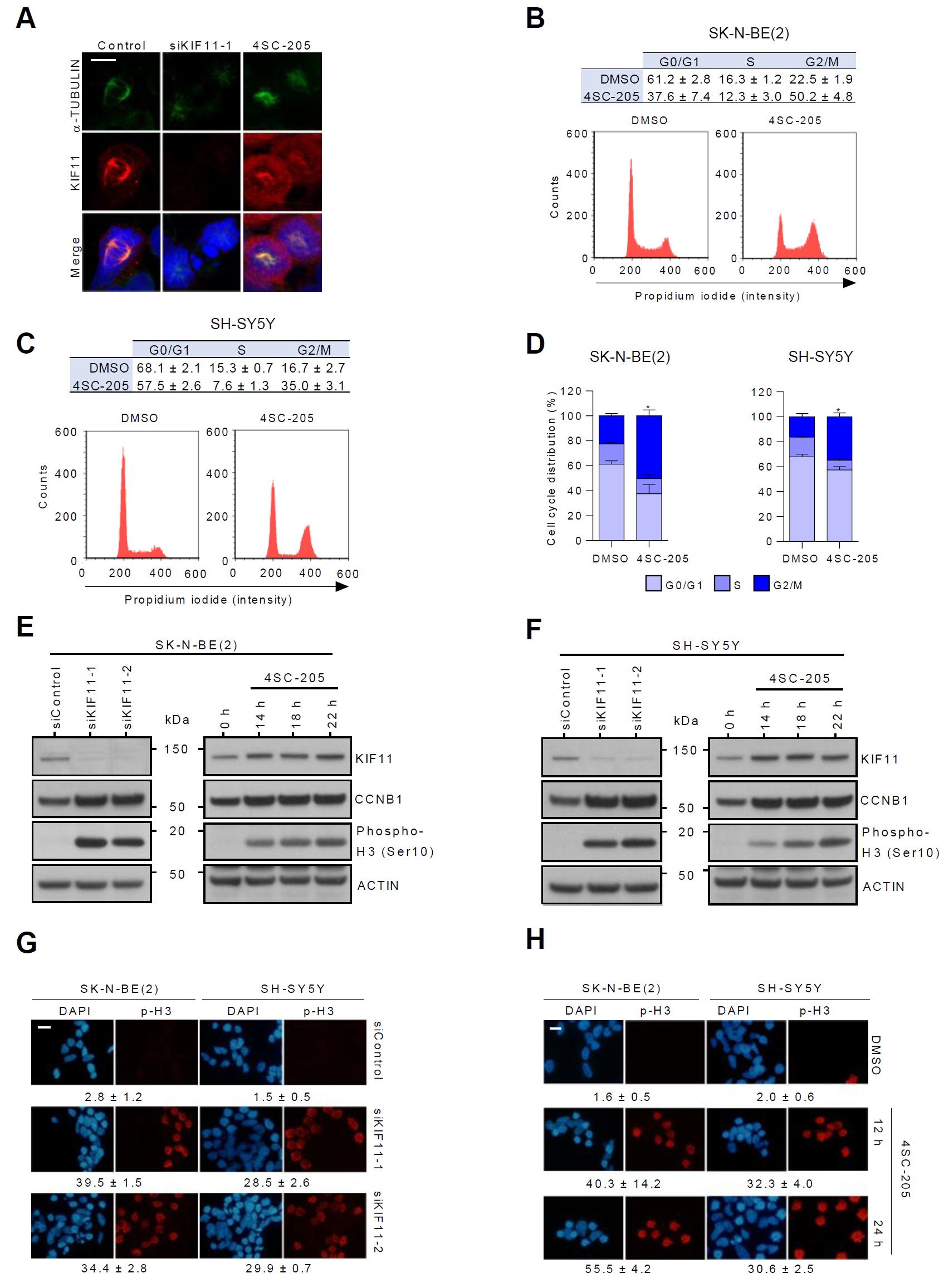


**Figure S4. Genetic and pharmacological inhibition of KIF11 reduces neuroblastoma cell proliferation and induces cell cycle arrest in mitosis. (A)** Mitotic spindle immunofluorescence of SH-SY5Y cells transfected with siKIF11 or treated with 4SC-205 (25 nM) for 24 h. KIF11: red, α-TUBULIN: green, DAPI: blue. Scale bar, 5 µm. (**B, C**) Cell cycle analysis of SK-N-BE(2) and SH-SY5Y cells treated with DMSO or 4SC-205 (25 nM) for 24 h analyzed by FACS. Histograms show one representative experiment from three independent experiments. (**D**) Graphs represent the average percentage of living neuroblastoma cells in G0/G1, S or G2/M phases of three independent experiments ± SEM. (**E, F**) Western blot analysis of KIF11 and cell cycle-related proteins CCNB1 and phospho-histone H3 at serine 10 in cells transfected with siControl, siKIF11 or treated with 25 nM 4SC-205 at the indicated time points. (**G, H**) Representative immunofluorescence images of phospho-histone H3 in the indicated neuroblastoma cell lines transfected with two independent KIF11-targeting siRNA (**H**) or treated with 4SC-205 at 25 nM (**G**). Scale bar, 10 µm. The percentage of phospho-histone H3 positive cells is shown below each condition and represents the average of 7 representative fields ± SEM. * *p* < 0.05, *** *p* < 0.001, two tailed Student’s t-test.


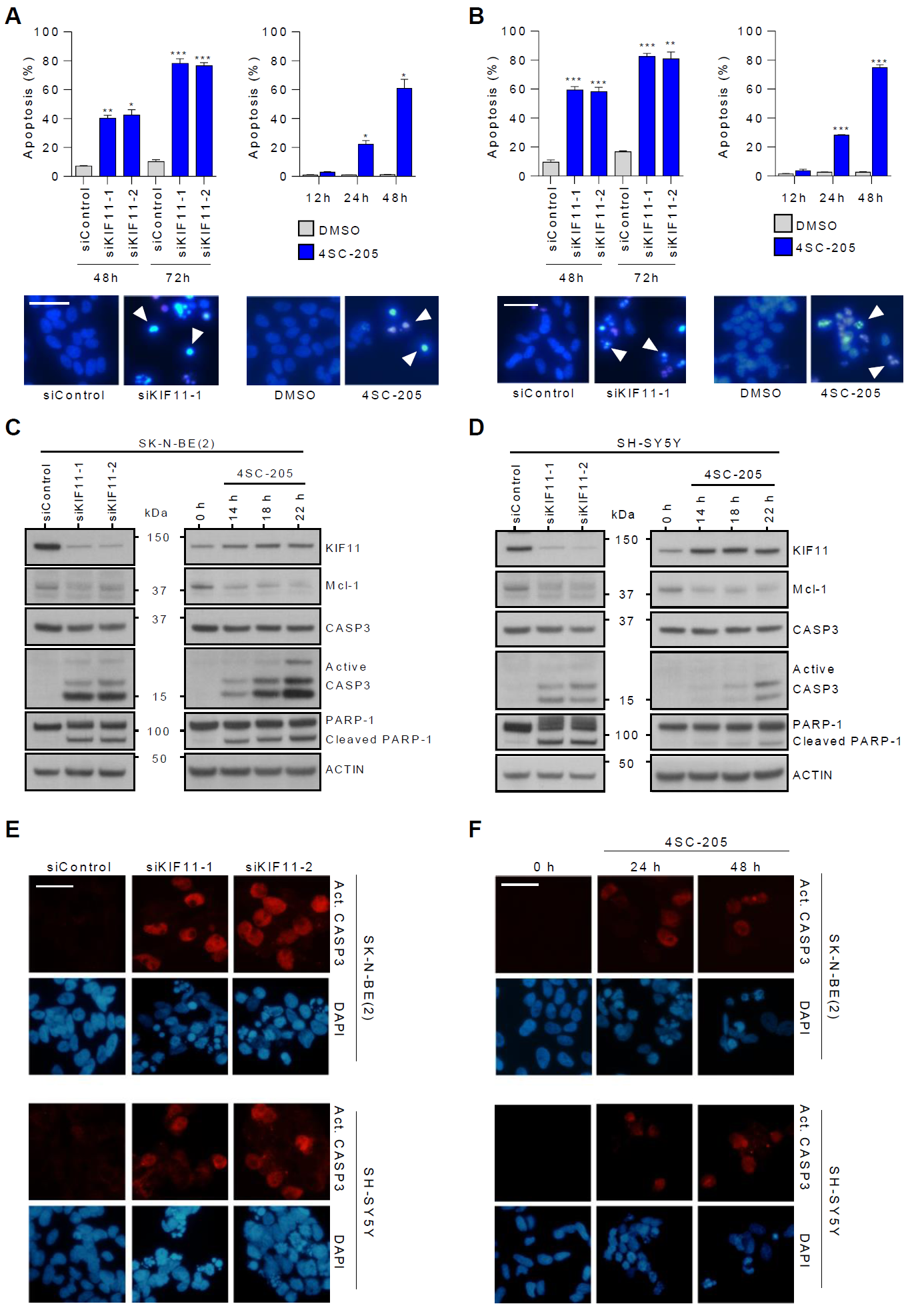


**Figure S5. Genetic and pharmacologic inhibition of KIF11 induces apoptosis.** (**A**, **B**) Quantification of apoptotic cells in SK-N-BE(2) (**A**) and SH-SY5Y (**B**) by Hoechst staining. Each graph represents the mean ± SEM of three independent experiments (n = 6 per experiment). * *p* < 0.05, ** *p* < 0.01, *** *p* < 0.001, two-tailed Student’s t-test. Lower panels show representative images of chromatin staining of neuroblastoma cells transfected with control siRNA (siControl) / siKIF11 or treated with 25 nM 4SC-205 for 48 h. White arrowheads point to cells with condensed and/or fragmented chromatin. Scale bar, 20 µm. (**C**, **D**) Western blot analysis of the indicated apoptosis-related proteins in SK-N-BE(2) and SH-SY5Y cells transfected with siControl/siKIF11 or treated with 4SC-205 (25 nM) at the indicated time points. (**E**, **F**) Active caspase-3 immunostaining of SK-N-BE(2) and SH-SY5Y cells transfected with siKIF11 (**E**) or treated with 25 nM of 4SC-205 (**F**) for 48 h. Scale bar, 20 µm.


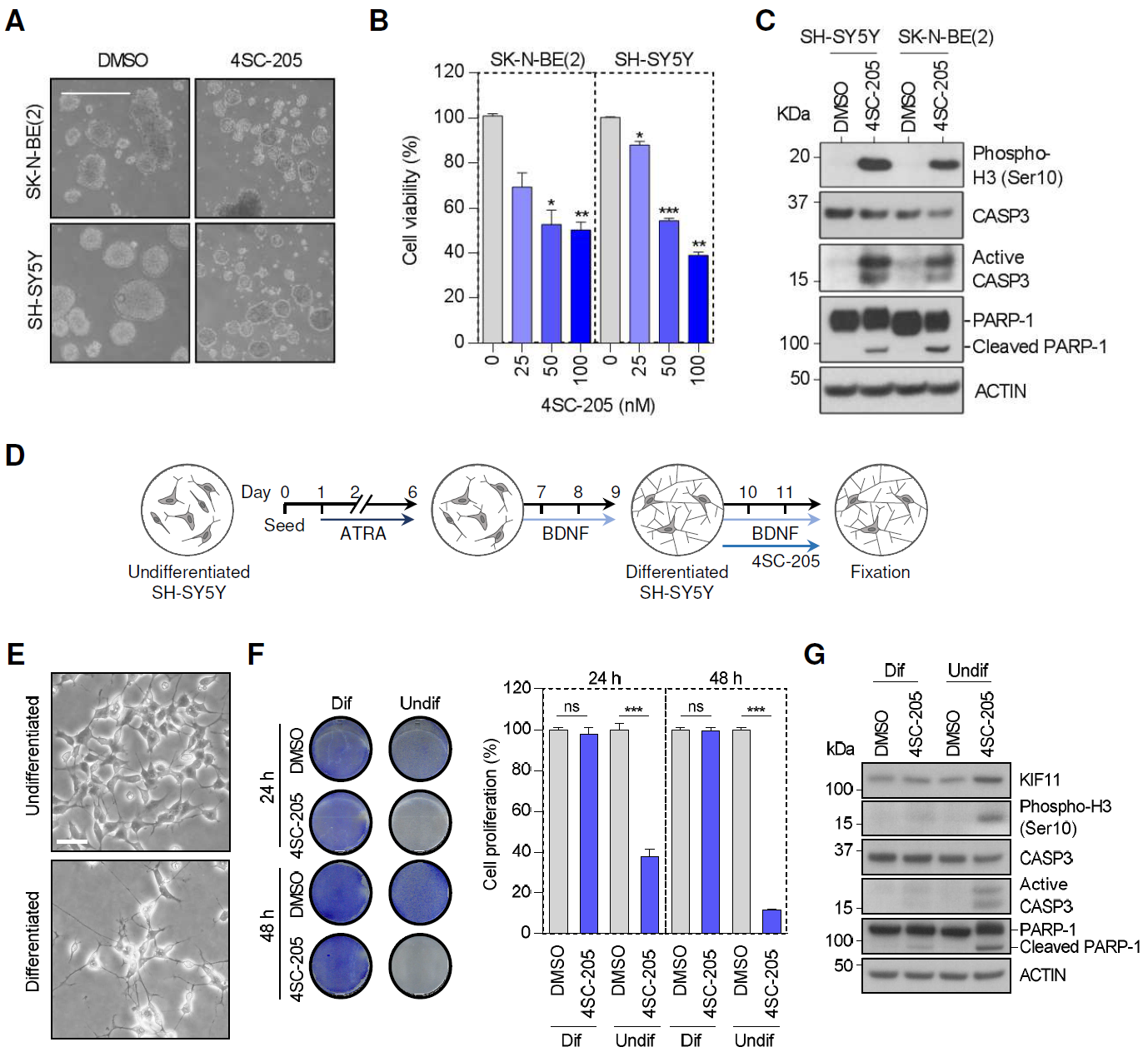


**Figure S6. Characterization of the 4SC-205 inhibitor.** (**A**) Representative images of three-dimensional (3D) spheroid culture of SK-N-BE(2) and SH-SY5Y. Scale bar, 100 µm. (**B**) Cell viability assay of neuroblastoma spheroids treated with DMSO or the indicated doses of 4SC-205 for 48 h. Cell viability was measured by MTS. Graphs represent the average of three independent experiments ± SEM * *p* < 0.05; ** *p* < 0.01; *** *p* < 0.001. (**C**) Western blot analysis of phospho-histone H3 at serine 10 and apoptosis-related proteins in SH-SY5Y and SK-N-BE(2) spheres treated with 25 nM 4SC-205. (**D**) Overview of the experimental design. (**E**) Representative images of SH-SY5Y cells before (upper image) and after 10 µM all-trans-retinoic acid / 50 ng/mL BDNF-induced differentiation (lower image). Scale bar, 50 µm. (**F**) Left, representative images of crystal violet staining. Right, cell proliferation of SH-SY5Y treated with DMSO or 4SC-205 in proliferating versus differentiated cells. Results are expressed as the average of three independent experiments (n = 2/condition) ± SEM. *** *p* < 0.001, two-tailed student’s t-test. (**G**) Western blot analysis of phospho-histone H3 and apoptosis-related proteins in differentiated and undifferentiated SH-SY5Y cells treated for 24 h with 25 nM 4SC-205.


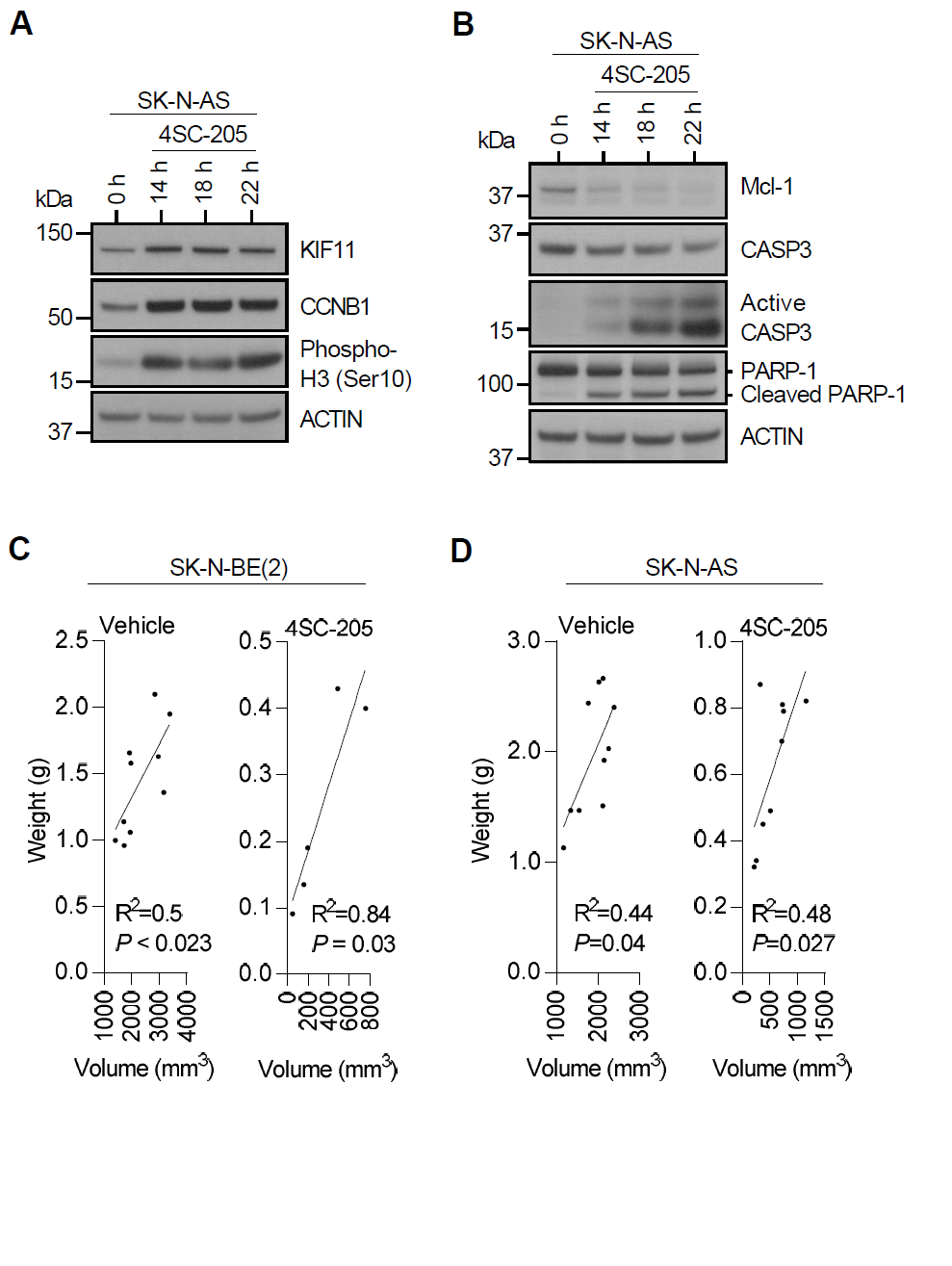


**Figure S7. 4SC-205 impairs tumor growth *in vitro* and *in vivo*.** (**A, B**) SK-N-AS cells were treated with 25 nM 4SC-205 at the indicated time points. Cell cycle-related genes (**A**) or apoptosis-related genes (**B**) were analyzed by western blot. (**C, D**) Correlation analysis between tumor weight and tumor volume in from SK-N-BE(2) and SK-N-AS subcutaneous xenografts.


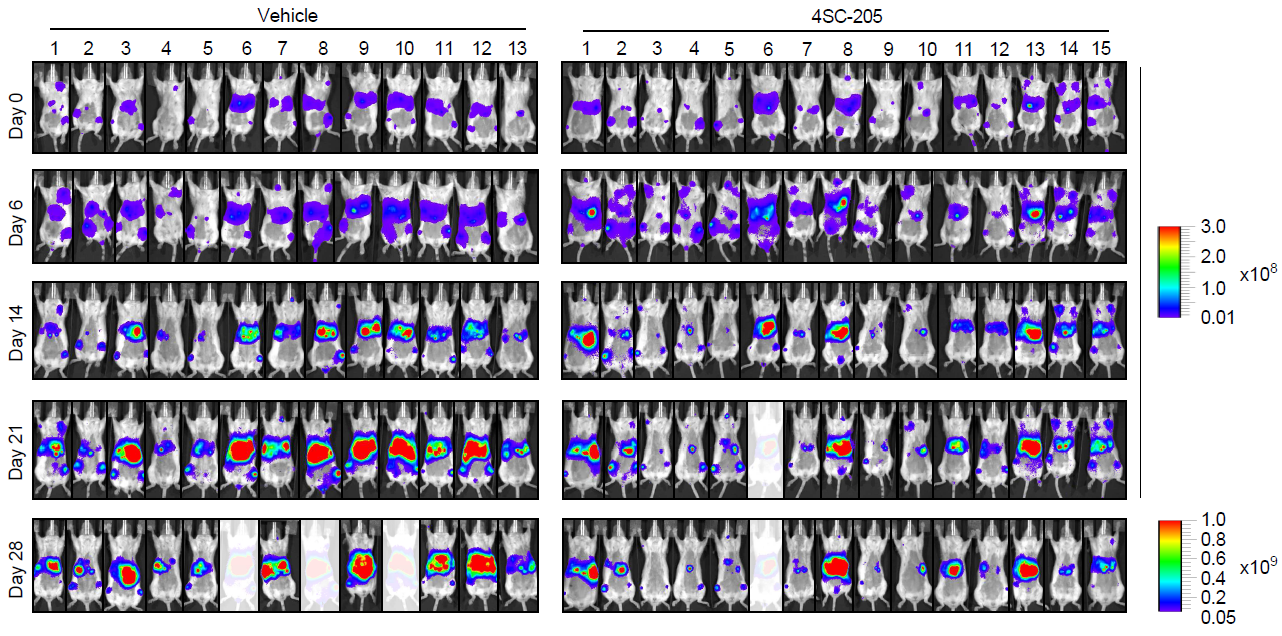


**Figure S8. 4SC-205 delays neuroblastoma liver metastases growth.** *In vivo* bioluminescence imaging of all mice bearing SK-N-BE(2) liver metastases at the indicated days. Mouse #6, #8, #10 from vehicle and #6 from 4SC-205 groups were euthanized before the last *in vivo* bioluminescence imaging.


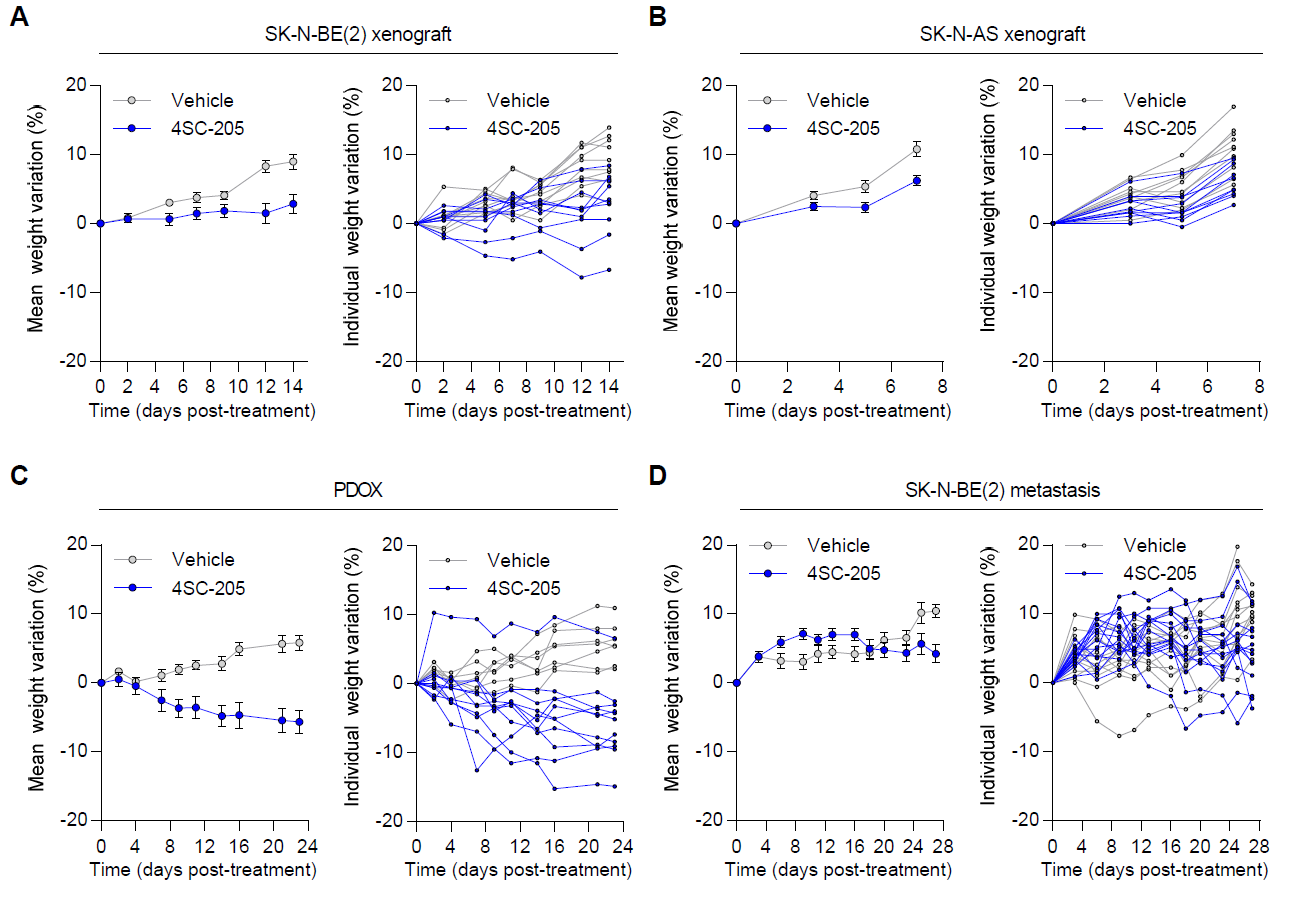


**Figure S9. 4SC-205 is well tolerated in mice**. Average (left panels) and individual (right panels) mouse weight variation during vehicle or 4SC-205 oral administration in SK-N-BE(2) (**A**), SK-N-AS (**B**), PDOX (**C**) or liver metastases (**D**) xenograft models.


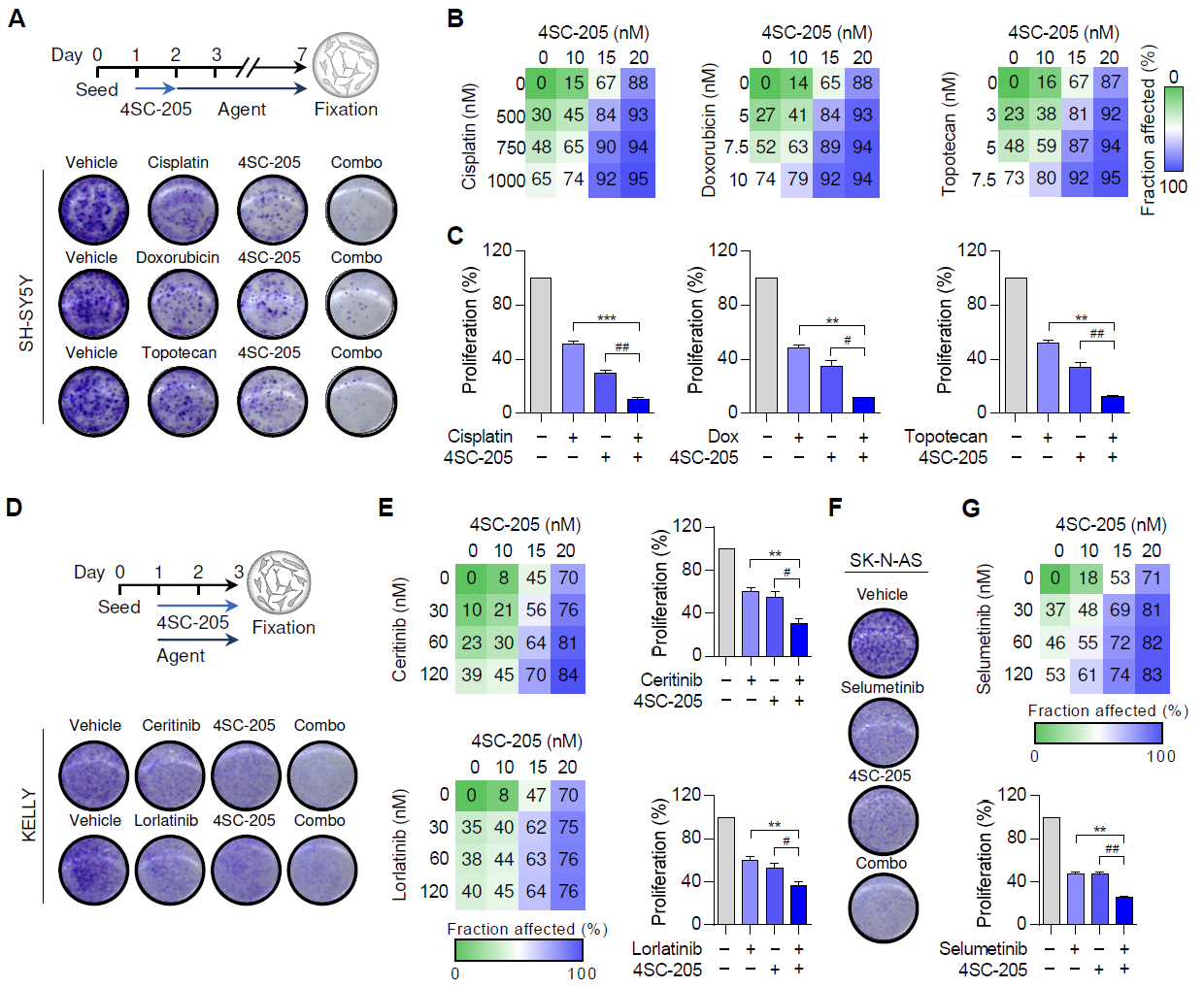


**Figure S10. 4SC-205 potentiates neuroblastoma therapies.** (**A**) Scheme of the experimental design for combination of 4SC-205 and standard chemotherapies. Representative images of SH-SY5Y cells treated with CDDP (750 nM), doxorubicin (7.5 nM), topotecan (5 nM), 4SC-205 (15 nM) and their corresponding combinations. (**B**) Heatmaps showing the percentage of the cellular fraction affected by the drug combination treatments. (**C**) Graphs represent the average effect on cell viability from of three independent experiments ± SEM (n = 2 /condition). (**D**) Schematic representation of the experimental design combining 4SC-205 and neuroblastoma targeted therapies. Images (lower panels) are representative of the ALK-mutated KELLY cell line treated with the ALK inhibitors ceritinib (120 nM) and lorlatinib (120 nM) alone or in combination with 15 nM 4SC-205 (Combo). (**E**) Heatmaps show the percentage of the cellular fraction affected by the drug combination treatments. Graph represents the average of three independent experiments (n = 2/condition) ± SEM. (**F**) The *NRAS*-mutated cell line SK-N-AS was treated with selumetinib (120 nM), 15 nM 4SC-205 or in combination (Combo) for 48 h. Images are representative of the crystal violet staining. (**G**) Heatmap shows the percentage of the cellular fraction affected by the drug combination treatments. Graph represents the average of three independent experiments (n = 3/condition) ± SEM. Selumetinib was used at 120 nM and 4SC-205 at 15 nM. * *p* < 0.05, ** *p* < 0.01, *** *p* < 0.001, two tailed Student’s t-test.

**Supplementary Tables**
